# Drug-related immune-mediated myelopathies

**DOI:** 10.3389/fneur.2022.1003270

**Published:** 2022-09-29

**Authors:** David Gritsch, Cristina Valencia-Sanchez

**Affiliations:** ^1^Division of Neuro-Oncology, Department of Neurology, Massachusetts General Hospital Cancer Center, Harvard Medical School, Boston, MA, United States; ^2^Department of Neurology, Mayo Clinic, Scottsdale, AZ, United States

**Keywords:** immune check inhibitor, myelopathy, TNF inhibitor, immune related adverse event, myelitis

## Abstract

Iatrogenic immune-mediated inflammatory disorders of the spinal cord are an uncommon but potentially severe complication of drug therapy for several human diseases. Particularly the introduction of novel biological agents in the treatment of systemic inflammatory disorders and cancer immunotherapy have led to a significant increase in immune-related adverse events of the central nervous system (CNS). The use of Tumor necrosis factor alpha (TNF-alpha) inhibitors in rheumatic and inflammatory bowel diseases has been associated with demyelinating and other inflammatory CNS conditions, including myelitis. The introduction of immune checkpoint inhibitors in the treatment of several human malignancies has led to an increase in drug-induced immune-related adverse events including in the CNS. Other drugs that have been associated with immune-mediated myelitis include tyrosine-kinase inhibitors and chimeric antigen receptor (CAR) T Cell therapy. A high degree of suspicion is necessary when diagnosing these conditions, as early diagnosis and treatment is crucial in preventing further neurological damage and disability. The treatment of drug-induced inflammatory myelitis typically involves administration of high-dose intravenous corticosteroids, however additional immunosuppressive agents may be required in severe or refractory cases. While most cases are monophasic and remit following discontinuation of the offending agent, chronic immunosuppressive therapy may be indicated in cases with a progressive or relapsing disease course or when a diagnosis of a specific underlying neuro-inflammatory disorder is made. Outcomes are generally favorable, however depend on the specific therapeutic agent used, the clinical presentation and patient factors. In this review we aim to describe the clinical characteristics, imaging findings and management for the most common forms of iatrogenic immune-mediated myelopathies.

## Introduction

There has been a significant rise in the number of reported cases of iatrogenic immune-mediated inflammatory disorders of the central nervous system (CNS). The development of novel biologic agents targeting the immune system has led to dramatic improvements in the treatment of certain autoimmune diseases and human malignancies (1–4). However, the increased use of these agents has also led to an increased risk of demyelinating and other inflammatory immune related adverse events (irAEs) of the CNS (5, 6). This includes worsening of pre-existing autoimmune diseases, *de novo* emergence of both T Cell and antibody mediated autoimmune disorders, and isolated inflammatory CNS events (3, 7, 8).

Particularly the introduction of tumor necrosis factor alpha (TNF-alpha) inhibitors (TNFIs) for the treatment of rheumatic disorders and inflammatory bowel diseases (IBD) and more recently the remarkable success of cancer immunotherapy, have been associated with an increase in iatrogenic inflammatory spinal cord disorders (3, 7, 8). Reports of drug-induced demyelination range from isolated monophasic events to multifocal disease fulfilling diagnostic criteria for multiple sclerosis (MS) and antibody mediated demyelinating disorders such as neuromyelitis optica spectrum disorder (NMOSD) and myelin oligodendrocyte glycoprotein-IgG (MOG-IgG) associated disease (MOGAD) (3, 5, 8). While drug induced inflammatory myelopathies are rare, they can be severe, and the incidence is likely to increase in the coming years as an increasing number of novel biologic agents find their way into clinical practice. In this literature review, we will therefore describe clinical characteristics, neuroimaging findings and therapeutic considerations for the most common forms of iatrogenic immune-mediated myelopathies. In addition, we will share representative cases of drug induced inflammatory myelopathies from our own experience at the Mayo Clinic.

## Tumor necrosis factor alpha inhibitors

TNFIs have proven to be highly effective for the treatment of certain autoimmune diseases, including rheumatoid arthritis (RA), psoriasis/psoriatic arthritis, ankylosing spondylitis and IBD (9–11). TNFIs currently approved by the US Food and Drug Administration (FDA) include etanercept, infliximab, adalimumab, golimumab, and certolizumab pegol (12). The use of TNFIs has been associated with a range of non-neurological and neurological side effects, including demyelinating and non-demyelinating inflammatory complications of the CNS and peripheral nervous system (PNS) (7, 12, 13). An association between TNFIs and demyelinating CNS disease has been well-documented in the literature (12, 13). An early phase I safety study of intravenous monoclonal anti-TNF antibodies in two patients with MS resulted in a transient increase in gadolinium-enhancing lesions on brain magnetic resonance imaging (MRI) and inflammatory abnormalities on cerebrospinal fluid (CSF) analysis (14). A subsequent double-blind, placebo-controlled phase II study of the TNFI lenercept in patients with relapsing-remitting MS found a significantly increased rate and severity of clinical exacerbations in the treatment group (15). Kumar et al. found in a literature review 56 cases of TNFI-induced central demyelination, reported between January 2008 and December 2018. Of these, 21 (37.5%) presented with myelitis, while the remainder presented with either optic neuritis, clinically isolated syndrome (CIS) or acute disseminated encephalomyelitis (ADEM) (13). Notably, none of the patients had pre-existing MS before starting treatment with TNFIs. Kunchok et al. performed a nested case-control study of patients with autoimmune diseases posing an indication for TNFIs and inflammatory CNS events between January 2003 and February 2019. The authors identified a total of 56 patients with inflammatory demyelinating CNS events, of which 39 (70%) were exposed to TNFIs compared with 28 (50%) in the control group. Interestingly, while most patients had MS or CIS, there were also 3 patients with aquaporin-4-IgG (AQP4) positive NMOSD, and one patient with MOG-IgG associated myelitis. In addition, the authors identified 50 patients with inflammatory non-demyelinating CNS events, consisting in meningitis, meningoencephalitis, autoimmune encephalitis, CNS vasculitis and neurosarcoidosis, including one case of sarcoid myelitis. Of the patients with inflammatory non-demyelinating CNS events, 25 (50%) were exposed to TNFIs compared with 14 (28%) of patients in the control group. The risk of any inflammatory CNS disease was significantly increased with TNFI exposure and when stratified by disease type, this association appeared to be predominantly observed in patients with RA (12). Additional evidence comes from the BIOGEAS Registry, a multicenter study led by the Study Group on Autoimmune Diseases (GEAS) of the Spanish Society of Internal Medicine. The registry reported a total of 803 cases of CNS demyelination in patients exposed to biologics, 92% of which occurred after exposure to TNFIs. Of 651 cases where the specific demyelinating syndrome was known, 254 were classified as MS-like, 504 as isolated optic neuritis, 17 as isolated myelitis, and 2 as neuromyelitis (16).

### Possible pathogenic mechanisms

Several possible mechanisms for TNFI-associated CNS inflammation have been proposed (12, 13, 17). TNF-alpha is a cytokine with important physiologic functions involved in the development and regulation of the immune system, pathogen and tumor defense, tissue regeneration and regulation of inflammation (17). Outside the CNS, TNF-alpha is produced predominantly by the effector cells of the immune system, and within the CNS by microglia, neurons and astrocytes (18, 19). Under pathological conditions, TNF-alpha acts as a proinflammatory cytokine that is thought to contribute to tissue inflammation and end organ damage in several autoimmune diseases, as outlined above (17). TNF-alpha exists in two forms that act on different receptors to fulfill somewhat opposed functions: Soluble TNF-alpha predominantly binds TNF type-1 receptor (TNFR1) and activates genes involved in apoptosis and chronic inflammation, while the transmembrane form of TNF-alpha predominantly binds TNF type-2 receptor (TNFR2) and promotes cell survival, anti-inflammatory effects and remyelination (13, 17). One hypothesis is that TNFIs may promote demyelination and inflammation by blocking TNF-alpha effects on TNFR2 in the CNS (13, 17). Other authors have postulated a paradoxical increase of TNF expression in the CNS, due to the inability of TNFIs to cross the blood brain barrier (BBB), an increase in autoimmunity due to reduced TNF-mediated survival and proliferation of regulatory T cells or downregulation of interleukin-10 and upregulation of interleukin-12 and interferon-γ (20, 21). Finally, an increased risk of demyelinating disorders in patients with rheumatic and inflammatory bowel diseases has been well-documented (22–24). However, the causal involvement of any single or a combination of the aforementioned mechanisms in TNFI induced inflammation remains currently unclear.

### TNFI-associated myelitis: Clinical presentation, diagnostic findings and treatments reported

**Figure 1:**
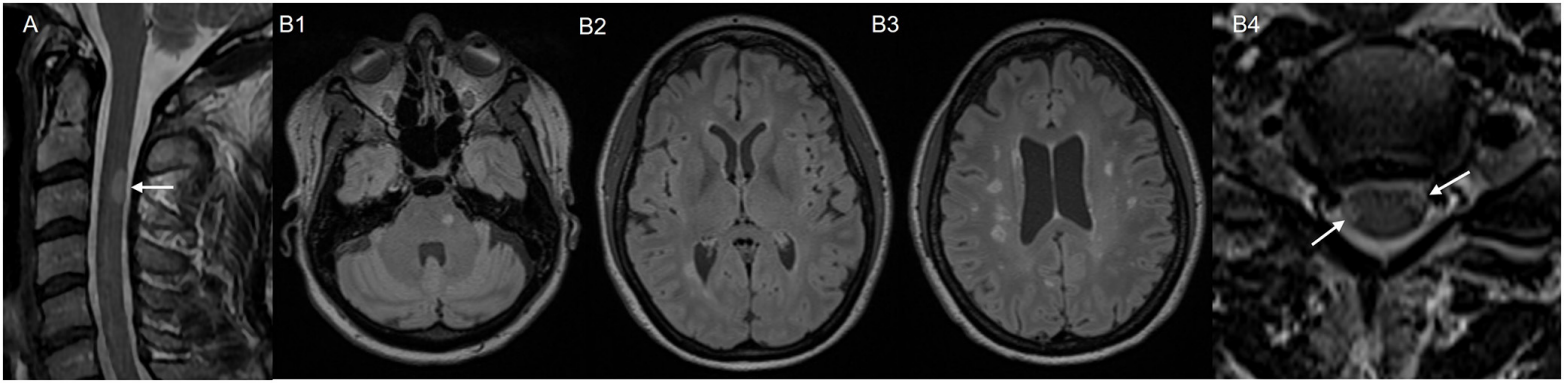
MRI images of two representative Mayo clinic cases. Case 1 with post-TNFI myelitis: **(A)** MRI cervical spine, sagittal T2 sequence, showing T2 hyperintense lesion in the posterior spinal cord at C3. Case 2 with post ICI-myelitis: **(B1–B3)** MRI brain, axial T2 fluid-attenuated inversion recovery (FLAIR) sequence, obtained prior to immune checkpoint inhibitor treatment showing multiple white matter T2 hyperintensities suggestive of demyelination. **(B4)** MRI cervical spine, axial T2 sequence, obtained after immune checkpoint inhibitor treatment, showing two spinal cord lesions suggestive of demyelination (arrows).

*Representative clinical case 1:* A 48-year-old male with a history of seronegative rheumatoid arthritis developed subacute numbness and weakness of the left upper extremity, and numbness of the left side of his chest. He had been on treatment with adalimumab for more than 10 years. He had previously been treated with methotrexate and etanercept. Of note, the patient's mother and grandmother had a history of multiple sclerosis. He underwent MRI of the cervical spine 5 months after onset of symptoms, which revealed a T2 hyperintense lesion at C3 (Figure 1A). MRI of the brain and thoracic spine were unremarkable. CSF analysis revealed CSF-unique oligoclonal bands (OCBs). He was diagnosed with CIS. TNFI was discontinued and he initiated methotrexate for arthritis. He was recommended clinical and radiologic monitoring for CIS. Two years after onset of symptoms he had not developed new demyelinating lesions.

The literature regarding TNFI-associated myelitis is currently limited to case reports and small case series. To the best of our knowledge, there are currently 21 reported cases with clinically and radiographically confirmed TNFI-associated myelitis (Supplementary Table 1) (25–38). The median age of the patients was 52 years with male to female ratio of 1:3. Of these, 11 patients received etanercept, 5 adalimumab, 4 infliximab, and 1 certolizumab with an exposure time ranging from 4 months to 8 years. The indication for TNFI use was RA and/or lupus in 9 patients, psoriasis and/or psoriatic arthritis in 5 patients, Crohn's disease in 3 patients, ankylosing spondylitis in 3 patients and necrobiosis lipoidica in one patient. The clinical presentation included paraparesis with sensory and motor deficits in 8 patients, isolated sensory deficits in 9, isolated motor deficits in 2, and unclear in 2 patients. Sphincter involvement was reported in 6 cases (25–38). MRI of the spine was positive for myelitis in 20 patients and negative in one patient despite a clinical picture that was highly suggestive of myelitis (33). MRI findings included T2-hyperintensities located in the cervical and/or thoracic cord with short segment monofocal distribution in 11 patients, longitudinally extensive in 7 patients, multifocal in 2 patients. Enhancement of the spinal lesions was observed in 7 of 12 cases where contrast was administered. CSF analysis performed in 19 cases demonstrated lymphocytic pleocytosis in 8 cases that was generally mild. CSF OCB testing was positive in 8/13 cases. MRI brain performed in 20 patients demonstrated signs of demyelination in 7 (25–38). TNFI was discontinued in all patients. Fourteen patients were treated with high-dose intravenous methylprednisolone (IVMP) for 3–5 days and one patient received rituximab. TNFIs were discontinued without additional acute therapy in 6 patients. Outcomes were reported as complete response in 5 patients, partial response in 11 patients and no response in 5 patients. Notably, all patients with complete response had received IVMP, while in the majority of patients without response TNFI discontinuation was the sole treatment (25–38). Six patients fulfilled clinical and/or radiographic criteria for new-onset MS at the time of presentation with myelitis, in 3 cases with subsequent MS relapses, and one patient developed progressive MS (25, 27, 35, 36). In addition, one patient developed MOGAD with longitudinally extensive myelitis (LETM) followed by optic neuritis (33). Flare up or continued disease activity of the underlying disorder was reported in 6 cases following discontinuation of TNFIs (26, 29, 31, 33, 35, 38), and these were generally treated with disease modifying therapies other than TNFIs (see Supplementary Table 1).

### Recommendations for treatment of TNFI-associated myelitis

No evidence-based recommendations or guidelines for the treatment of TNFI-associated myelitis are currently available. Acute treatment is extrapolated from the experience with other inflammatory/demyelinating CNS diseases and often involves high-dose IVMP. TNFIs are typically discontinued once myelitis is suspected clinically or diagnosed via neuroimaging (12, 13, 17, 39). Given the association with worsening of demyelinating disease, TNFIs should generally be avoided in patients with pre-existing MS or other demyelinating diseases (13, 17, 39–41). The safety of reinitiating TNFI therapy is currently unclear. Flare up or continued disease activity of the underlying rheumatic or inflammatory bowel disorder occurred in a quarter of the patients after discontinuation of TNFI, requiring disease modifying therapy, although none of these patients were rechallenged with TNFI. Recurrence of demyelination with TNFI rechallenge has been described in the literature, however appears to be rare (17, 26, 42). Reintroduction of TNFIs may therefore be safe in selected cases with isolated myelitis and good response to acute therapy and in the absence of an underlying demyelinating disease (17, 42). A relapsing-remitting course should prompt further investigation regarding an underlying disease process including relapsing-remitting MS, AQP4+NMOSD and MOGAD. Long-term immunomodulatory therapy may be required in these cases and should take into consideration the specific demyelinating disorder, as well as treatment of the coexisting autoimmune disorder that the TNFI was used for (13, 39). The outcome of TNFI-associated myelitis appears to be generally favorable with most patients achieving a partial or complete remission following acute treatment (12, 13, 17, 26). However, refractory and recurrent cases do exist and continue to pose a therapeutic challenge.

## Immune checkpoint inhibitors

Immune checkpoint inhibitors (ICIs) have been used successfully in the treatment of several systemic malignancies including advanced melanoma, renal cell carcinoma, head and neck cancers and non-small cell lung cancer (NSCLC). ICIs in current clinical use can be categorized by their molecular target into one of the following three categories: programmed cell death receptor-1 (PD-1) inhibitors (nivolumab, pembrolizumab, and cemiplimab), programmed cell death ligand-1 (PDL-1) inhibitors (atezolimumab, durvalumab, and avelumab), and cytotoxic T-lymphocyte-associated antigen-4 (CTLA-4) inhibitors (ipilimumab) (1, 4, 43). Under physiologic conditions, immune checkpoints are molecular breaks that limit lymphocyte activation in response to antigenic stimuli and thereby aid in the prevention of autoimmunity (44, 45). Cancer cells are able to exploit this mechanism and evade the immune system by inducing activation of lymphocyte immune checkpoint molecules (43). ICIs stimulate the anti-tumoral immune response by inhibiting immune checkpoints and thereby leading to increased immune cell activation in response to cancer-antigens (46). While ICIs have been proven successful as anticancer agents, their use has also been associated with several irAEs, which can involve several organ systems, most commonly the gastrointestinal tract, skin, endocrine organs and liver (47). Although most irAEs are mild, severe adverse events can occur and frequently require discontinuation of ICIs and immunosuppressive treatment (48). Neurologic immune-related adverse events (nAEs) are rare and have traditionally been reported to occur in <1% of patients, however, they can potentially be severe and disabling. The range of nAEs is broad and may affect any level of the peripheral and the central nervous system, with neuromuscular complications being the most common (3, 49). CNS nAEs are usually severe (grade ≥ 3) and can be associated with a high fatality rate (6.3–12.8% for encephalitis; 7.4–8.3% for meningitis) (50, 51). Several cases of ICI-related demyelination, including worsening of preexisting MS or *de novo* diagnosis of MS, have been reported in the literature (8, 52). A recent review of ICI induced nAEs identified 428 reported individual patients across 256 articles. Demyelinating diseases comprised 4% of the reported cases, and half of them presented with myelitis (3). Oliveira et al. recently reported the results of a systematic literature review identifying 23 published cases of ICI-related CNS demyelination. The clinical spectrum of ICI induced demyelination included myelitis in 7 patients, optic neuritis in 4 patients, relapse of preexisting MS in 3 patients, progression of MS from radiographically isolated syndrome (RIS) in 2 patients and *de novo* NMOSD in one patient (8).

### Possible pathogenic mechanisms

Multiple possible pathogenic mechanisms for ICI induced irAEs have been discussed in the literature, including molecular cross-reactivity between tumor neoantigens and surface antigens found on normal cells, activation of “dormant” autoimmune diseases by clonal expansion of pre-existing self-reactive T cells, increased production of pro-inflammatory cytokines due to a shift toward Th1/Th17 mediated immune response over Th2 activity, and induction or stimulation of B cell activity leading to autoantibody production (53–55). While the exact pathogenetic mechanisms that lead to ICI induced autoimmunity are currently unclear, two commonly cited theories include potentiation of autoimmune paraneoplastic disorders and exacerbation of pre-existent autoimmune diseases. It has been proposed that ICI treatment can contribute to the development of paraneoplastic disorders by induction or exacerbation of immune responses against shared autoantigens between the tumor cells and normal neural tissue (56). This is supported by results from an animal model of paraneoplastic cerebellar degeneration, where the disease could be induced by co-administration of antigen specific lymphocytes and a CTLA4 inhibitor in genetically altered mice expressing a shared antigen on Purkinje cells and cancer cells (57). This observation is further supported by several case reports, describing the emergence of paraneoplastic disorders in patients treated with ICIs (58). In addition, it has been suggested that ICI treatment induced immune activation may unmask dormant autoimmune disorders that previously remained subclinical (56, 59). This is supported by observations from animal studies showing that the course of experimental autoimmune encephalitis (EAE) is exacerbated in PD-1 deficient mouse models or in mice that were treated with an anti-CTLA4 monoclonal antibody (60, 61). In addition, there are several reports in the literature of patients treated with ICIs that developed de-novo autoimmune disorders including MS, NMOSD and MOGAD (8, 62–64). Oliveira et al. noted that of four cases with ICI induced CNS demyelination that underwent histopathological evaluation, all had CD8+T cell predominant infiltrates, suggestive of a Th1 immune response (8). In one of these cases, the authors compared functional profiling of the patient's myelin-reactive CD4+ T cells and identified an inflammatory Th1/Th17 phenotype that was similar to the one of myelin-reactive T cells in patients with MS (64). In addition to T cell activation, ICIs have also been demonstrated to influence B cell function and lead to increased production of antibodies, including pathogenic autoantibodies (55, 65, 66). Interestingly, Das et al. demonstrated that combined ICI treatment but not monotherapy, lead to an early increase in the circulating B cell population, as well as cytokine markers of B cell activation, and this was associated with an increased risk for grade 3 or higher irAEs 6 months after initial treatment (65). De Moel et al. evaluated the pre- and posttreatment sera of 133 ipilimumab-treated melanoma patients for 23 common clinical autoantibodies and found seroconversion in 19.2% of previously autoantibody negative patients (67).

It has been suggested that underlying immune dysregulation may be associated with a higher risk for developing irAEs, and therefore some studies have investigated cytokine profiles that could predict a higher risk of developing irAEs. One study identified lower baseline levels of CXCL9, CXCL10, CXCL11, and CXCL19, and greater increases in CXCL9 and CXCL10 levels after ICI treatment in patients with irAEs (68). In another study, higher levels of 11 cytokines (G-CSF, GM-CSF, Fractalkine, FGF-2, IFNα2, IL12p70, IL1a, IL1B, IL1RA, IL2, and IL13) was associated with severe irAEs (69). Other proposed biomarkers include increased soluble CD163 (70), higher pre-and post-treatment levels of soluble CTLA-4 in patients treated with ipilimumab (71), elevated pre and post-treatment levels of IL17 (72), low baseline levels of IL6 (73), and increased IL6 levels after ICI treatment (74). All these possible biomarkers require validation with additional larger studies. Identification of reliable biomarkers that could predict risk for development of irAE prior to ICI treatment would allow for better selection of patients and improve safety.

### ICI-associated myelitis: Clinical presentation, diagnostic test findings and treatments reported

*Representative clinical case 2*: A 50-year-old female was diagnosed with melanoma stage IIIA. She underwent excision followed by treatment with pembrolizumab. She underwent a brain MRI for tumor staging, which showed no metastases, but revealed multiple T2-hyperintense white matter lesions typical for demyelination located in periventricular, juxtacortical and infratentorial areas. Some of the lesions had T1 hypointensity suggesting chronicity (Figures 1B1–B3). The patient had never experienced prior episodes of focal neurological deficits suggestive of MS relapses. Three months after initiation of pembrolizumab, she developed severe arthralgias and she was diagnosed with seronegative inflammatory arthritis triggered by ICI. Pembrolizumab was discontinued and she was treated with oral prednisone and methotrexate with improvement of symptoms. Around the time that the arthralgias started, she also developed weakness of her right lower extremity and walking difficulties, which were initially thought to be related to arthritis. Symptoms partially improved after ICI discontinuation and prednisone, but she was eventually referred to Neurology 6 months later due to persistence of right lower extremity weakness. On exam she had pyramidal weakness of the right lower extremity, hyperreflexia, and extensor plantar response. Spine MRI revealed multiple short-segment, peripherally located, cervical and thoracic spinal cord lesions consistent with demyelination (Figure 1B4). CSF analysis revealed CSF-unique oligoclonal bands. Based on MRI and CSF findings, patient met criteria for MS diagnosis. Treatment with methotrexate was switched to rituximab to treat both inflammatory arthritis and MS.

Similar to TNFIs, our current knowledge about ICI-associated myelitis is largely limited to case reports and other anecdotal evidence. There are currently at least 30 cases of clinically and radiographically confirmed ICI-associated myelitis that have been reported in the literature (Supplementary Table 2) (62, 63, 75–95). The median age at onset was 61 years with a male to female ratio of 1.6:1. Of these, 8 patients received pembrolizumab, 6 ipilimumab, 6 nivolumab, 4 combination therapy with nivolumab and ipilimumab, 3 durvalumab, 2 atezolizumab and one patient received nivolumab and ipilimumab followed by pembrolizumab. The time to onset from ICI exposure to myelitis ranged from 2 weeks to 12 months. The primary cancer diagnosis was melanoma in 12 patients, NSCLC in 11 patients, small cell lung cancer (SCLC) in 3 patients, and Hodgkin lymphoma, transitional cell cancer of the bladder, clear cell renal cell carcinoma and mesenteric inflammatory myofibroblastic tumor in one patient each. A majority of patients presented with paraparesis, sensory deficits as well as bowel and/or bladder dysfunction. MRI of the spine was performed in 29 patients and demonstrated myelitis in all but one patient, where it was unrevealing despite clinical and CSF findings that were highly suggestive of myelitis (96). Notably, spinal MRI demonstrated longitudinally extensive lesions (LETM) in 20 patients, monofocal short-segment lesions in 4 patients, and multifocal lesions in 4 patients. MRI spine with contrast performed in 19 cases revealed enhancement of spinal lesions in 17 cases, and isolated nerve root enhancement in one case. CSF analysis available in 28 cases was abnormal in all but 3 cases, with mild to moderate lymphocytic pleocytosis being the most common finding. CSF OCB testing was reported in 11 cases and positive in 5. MRI brain was reported in 17 cases and demonstrated possible signs of demyelination in 5 cases (T2 hyperintensities of the brainstem in 2 cases, periventricular lesions with or without contrast enhancement in 3 cases) (62, 63, 75–95). Autoantibody testing from serum and/or CSF was reported in 19 cases and positive in 7, including: one case of CRMP5-IgG associated myelopathy following atezolizumab for SCLC, one case of AQP4-IgG in a patient receiving pembrolizumab for NSCLC and one patient receiving nivolumab for NSCLC, one positive low titer MOG-IgG and multiple paraneoplastic antibodies (CV2, SOX1, ZIC4) in a patient receiving durvalumab for SCLC, one case of GFAP-IgG in a patient receiving pembrolizumab for NSCLC, and two cases were reactivity to unknown neural antigens was identified on rodent sections (62, 63, 75, 76, 93). In 4 cases, myelitis occurred in conjunction with other irAEs (82, 89–91). All but one patient received treatment with steroids (high-dose IVMP 26, oral steroids 3), and the remaining patient was treated with intravenous immunoglobulin (IVIG) alone. Additional treatments included: plasmapheresis (10), additional IVIG (3), cyclophosphamide (3), infliximab (3), tocilizumab and ruxolitinib (1), methotrexate (1), rituximab (1). Recovery was partial in 17 cases, complete in 5 cases, minimal in 4 cases and absent in 3 cases. One patient had progressive myelitis despite treatment, and 9 patients suffered clinical and/or radiographic recurrence (62, 63, 68–88).

### Recommendations for diagnostic workup and management of ICI-associated myelitis

While treatment recommendations are largely based on anecdotal evidence, multiple societies have published guidelines for the management of irAEs including nAEs. The current guidelines from the American Society of Clinical Oncology (ASCO) recommend that the initial evaluation of patients with suspected nAEs should include a thorough workup for other secondary causes of neurological symptoms including cancer progression, infection, metabolic causes and other autoimmune disorders such as paraneoplastic syndromes and autoimmune encephalitides. MRI brain and/or spine with and without contrast and CSF analysis including cytology to detect cancer cells is recommended for most patients with suspected nAEs (97). For transverse myelitis the guidelines recommend neurological consultation, MRI of the spine with thin axial cuts, MRI of the brain with and without contrast, CSF analysis including cell count, protein, glucose, OCBs, viral PCRs, cytology and onconeural antibodies, serum studies including B12, HIV, rapid plasma reagin, ANA, Ro/La, TSH, and AQP4-IgG (98).

Differential diagnosis to consider in myelitis cases include spinal cord metastasis and radiation induced myelopathy. In a systematic review which included 7 patients with myelitis, 4 of the patients had been exposed to spinal irradiation for bone metastases before or during treatment with ICIs. However, it was noted that the dose of radiation was under 30 Gray (Gy) in all cases and the temporal course, neuroimaging findings and/or CSF findings were more suggestive of a demyelinating process (8).

ASCO treatment recommendations include permanent discontinuation of ICIs, administration of methylprednisolone 2 mg/kg with strong consideration for higher doses of 1 g/day for 3–5 days and IVIG treatment (98). Guidelines from the National Comprehensive Cancer Network (NCCN) for the treatment of ICI-induced transverse myelitis recommend methylprednisolone 1 g/day for 3 to 5 days with strong consideration for IVIG or plasmapheresis (99). Additional international guidelines come from the Society for Immunotherapy of Cancer (SITC) (100). While these guidelines do not specifically comment on the management of myelitis, they do recommend treatment with high-dose IVMP for severe nAEs with consideration for addition of IVIG (2 g/kg in divided doses over the course of 5 days), plasmapheresis (one session every other day for 5–7 cycles), or rituximab (375 mg/m^2^ weekly infusion for 4 weeks) in refractory cases (100). Guidelines from the European Society for Medical Oncology (ESMO) recommend discontinuation of ICIs for all but mild (grade 1) neurological symptoms (48). Additionally, treatment recommendations include prednisolone 0.5–1 mg/kg for moderate nAEs and oral prednisolone (1–2 mg/kg) or intravenous equivalent for significant neurological toxicity (48).

Most cases of nAEs appear to be monophasic and submit with discontinuation of ICIs and immunosuppressive therapy. However, prolonged corticosteroid treatment for several weeks may be reasonable in cases of ICI associated myelitis given the long half-life of ICIs (101). Our own approach is to use IV methylprednisolone 1,000 mg daily for 5 days, generally followed by 1,000 mg weekly for 6–12 weeks for patients with CNS involvement (except for mild meningitis). Long-term immunosuppressive therapy may be required in cases with progressive or relapsing disease courses or when a specific pathogenic antibody is detected that could suggest an underlying autoimmune disorder (62, 63, 75, 76). While the long-term prognosis in ICI-induced myelitis is currently unclear, the high rate of longitudinally extensive disease likely indicates a significant degree of neurological impairment in the affected patients. Additional research is required to identify patients at high risk for a refractory or recurrent disease course that would benefit from early initiation of plasmapheresis or more aggressive immunosuppressants. The group of myelitis triggered by ICI likely includes cases with different underlying pathophysiology, which might play a role in prognosis, and we speculate that ICI-triggered paraneoplastic myelopathies (such as the case associated with CRMP-5 IgG), might have worse prognosis than ICI-triggered demyelinating myelitis. In addition, ICI-triggered AQP4+ myelitis might have worse prognosis than those cases that resemble MS lesions (short segment myelitis), although this requires further research. Generally, ICIs are discontinued permanently in the setting of severe nAEs. Successful rechallenge with the same or a different class of ICIs have occasionally been reported, but this decision needs to be made on an individual basis. No strategies for prevention of nAEs currently exist, however it is recommended to avoid the use of ICIs in some patients with preexistent autoimmunity, particularly pre-existing paraneoplastic neurologic syndromes, or previous severe irAEs.

## Other drug classes associated with immune-mediated disorders of the spinal cord

### Tyrosine-kinase inhibitors

BCR-ABL tyrosine kinase inhibitors (TKIs) have revolutionized the treatment of specific types of cancer, including certain hematological malignancies and gastrointestinal stromal tumors (102–104). Neurological side effects with TKIs have previously been reported and these are generally mild and non-specific and include dizziness, insomnia, anxiety and depression (103, 104). However, more recently there have been several case reports of CNS demyelination, optic neuritis and transverse myelitis in patients treated with TKIs (104–106). Rotstein et al. described a patient who presented with subacute onset bilateral vision loss and sensorimotor paraparesis with urinary retention 8 weeks after being started on imatinib for hypereosinophilic syndrome. MRI of the spine demonstrated multiple mildly expansile and enhancing lesions of the cervical and thoracic cord. MRI brain was notable for increased T2 signal in bilateral optic nerves without contrast enhancement and a solitary non-enhancing white matter lesion. AQP4-IgG and MOG-IgG serum cell-based assays were negative. Imatinib was stopped and the patient received high-dose corticosteroids without response, followed by plasmapheresis. The patient subsequently gradually regained full visual acuity in both eyes and full strength in his lower extremities, with MRI brain and spine at 6-month follow-up showing significant radiographic improvement (106). Rafei et al. reported the case of a patient who presented with rapidly progressive sensorimotor deficits after being treated with imatinib for 15 years for chronic myelogenous leukemia (CML). MRI spine showed a T2 hyperintense lesion of the thoracic cord at T6–T7. Imatinib was discontinued and symptoms improved with high-dose steroids. Notably, the patient experienced clinical recurrence 1 year later after being started on dasatinib (104). Finally, Abuzneid et al. reported a patient that received imatinib for CML and presented with back pain, sensorimotor deficits and loss of sphincter tone. MRI spine demonstrated T2-hyperintensity extending from T6–T12. CSF analysis demonstrated lymphocytic pleocytosis. However, it should be noted that the patient had recently undergone total body irradiation and his myelitis had therefore been felt to represent radiation induced transverse myelitis. The patient received IVMP with a good partial response followed by plasmapheresis (105). While the exact mechanism of TKI induced neuroinflammation is currently unclear, it has been proposed that imatinib potentiates antitumoral immune responses by activation of CD8+ T cells and downregulation of T-regulatory cell responses, which may potentially contribute to immune-related effects (107). Currently there is not sufficient evidence to firmly tie the use of TKIs to the development of transverse myelitis or CNS demyelination in general and further research will be needed to confirm these initial observations.

### Chimeric antigen receptor T (CAR-T) cells

CAR-T cell therapy has proven to be highly effective for the treatment of certain types of leukemia and lymphoma and is currently under investigation for the treatment of several other malignancies (108, 109). However, the use of CAR-T has been associated with specific side effects including cytokine release syndrome (CRS) and a specific type of neurotoxicity that has been termed immune effector cell related neurotoxicity syndrome (ICANS) (110). Signs and symptoms of ICANS are variable, however often include a stereotyped presentation with encephalopathy, aphasia, incoordination, muscle weakness, numbness, hallucinations, myoclonus or tremor and this can progress to generalized seizures, coma and death (109). While CAR-T therapy has not typically been associated with demyelination, rare reports of CAR-T associated myelopathy have recently emerged (108, 111). Beauvais et al. reported a patient who developed myelitis following treatment with anti-CD19 CAR-T therapy for refractory diffuse B cell lymphoma. The patient developed grade 2 CRS on day 3 following CAR-T infusion, and this subsequently progressed to severe ICANS at day 5 with obtundation that required mechanical ventilation. MRI brain demonstrated linear T2 hyperintensities of the parietal white matter without contrast enhancement. The patient received treatment with IVMP with significant initial improvement of neurological symptoms, however on day 9 the patient developed sensorimotor tetraparesis. MRI spine demonstrated a longitudinally extensive T2-hyperintense spinal cord lesion from C2 to T11 without associated contrast enhancement. The patient received treatment with IVIG and anakinra, with near complete radiographic regression of the spinal cord lesion and partial clinical improvement with residual spastic paraparesis at 1 year follow-up (108). Nair et al. recently reported two patients with no known prior neurological disease who developed acute leukoencephalomyelopathy with quadriparesis following treatment with axicabtagene ciloleucel CAR-T therapy for B cell lymphoma. Both patients had developed moderate to severe ICANS followed by rapidly progressive paraparesis. MRI of the brain and spine demonstrated symmetric extensive supratentorial white matter T2 hyperintensities and holocord involvement in the first patient, and diffuse leptomeningeal supra- and infratentorial enhancement and spinal T2 hyperintensity running from C2–C4 in the second patient. CSF analysis was notable for elevated protein only in the first patient and mild leukocytic predominant pleocytosis in the second. Both patients received high-dose IVMP and tocilizumab and made a partial recovery (111). CAR-T related myelopathy is an extremely rare and unusual manifestation of CAR-T neurotoxicity and only very limited data regarding clinical features and optimal management exist in the literature. Nevertheless, CAR-T related myelopathy should be considered as a differential diagnosis when patients present with clinical signs and symptoms of myelitis following CAR-T infusion.

## Discussion

Iatrogenic immune-mediated neurological disorders are an important complication of modern pharmacotherapy. While generally considered uncommon, the introduction of novel immunomodulatory agents in the treatment of several conditions has led to a significant increase in reports of immune-related adverse events of the CNS. Particularly the rise of cancer immunotherapy with ICIs and more recently CAR-T therapy has led to increased awareness for nAEs.

Drug-induced transverse myelitis is a rare but potentially severe complication of treatment with TNFIs and certain cancer treatments, particularly ICIs. There are some differences in the clinical features and diagnostic findings that likely reflect the different underlying pathogenesis of these drug induced myelitis (Table 1). Time till onset of myelitis appears to be shorter in ICI treated patients ranging from weeks to months, whereas TNFI treated patients often received the drug for months to several years before onset of symptoms. Based on the currently available case reports it appears that longitudinally extensive disease is common in ICI associated myelitis and less frequently seen with TNFIs. These differences could be related to a stronger effect of ICI enhancing immune responses which could lead to early and more severe inflammatory complications. CSF pleocytosis has been reported more commonly with ICI myelitis, while CSF-unique OCBs have been reported more frequently with TNFI associated myelitis, although the exact pathogenetic and potential treatment implications of this finding remains currently unclear. More aggressive immunosuppressive treatments were used for ICI myelitis acutely, possibly due to the high rate of longitudinally extensive disease. A significant proportion of cases with TNFI associated myelitis were treated with discontinuation of TNFI alone, while high-dose corticosteroids were used in most patients with ICI associated myelitis and treatment with IVIG, plasmapheresis and other immunosuppressant agents was common. Although many patients with drug-associated myelitis achieved partial or complete recovery with discontinuation of the offending agent and/or immunosuppressive therapy, treatment refractory cases have been reported. In TNFI treated patients, occurrence of new demyelinating CNS events and progression to MS appears to be fairly common. In ICI treated patients, complications included irAE affecting other organ systems and recurrence of myelitis.

**Table 1:** Summary of main clinical and diagnostic findings in myelitis associated with TNFI and ICI.

	**TNFI (*n* = 21)**	**ICI (*n* = 30)**
Mean age (SD)	52 (7.87)	61 (12.61)
M:F	1:3	1.6:1
Time treatment initiation to neurological symptom onset	4 to 96 months	0.5 to 12 months
LETM	33% (7/21)	67% (20/30)
MRI contrast enhancement	58% (7/12)	89% (17/19)
MRI brain abnormality	35% (7/20)	29% (5/17)
CSF pleocytosis	42% (8/19)	75% (21/28)
CSF-unique OCBs	62% (8/13)	46% (5/11)
Autoantibodies positive	20% (1/5)	37% (7/19)
Improvement after treatment (partial or complete)	76% (16/21)	73% (22/30)

F, female; LETM, longitudinally extensive transverse myelitis; M, male; OCB, oligoclonal bands.

The broad spectrum of clinical manifestations suggests that diverse pathophysiological mechanisms are likely involved. For instance, the association between TNF inhibition and CNS demyelination has been well-documented in the literature. However, the association of TNFI and ICI with disorders such as MOGAD or NMOSD is less clear, and these antibodies have been infrequently reported (112). In particular, TNF alpha levels have been shown to be elevated in CSF in patients with AQP4 and MOG IgG autoimmunity, but not in patients with MS, and therefore the effect of TNF inhibition is likely different in these CNS inflammatory disorders (113). A significantly different mechanism also needs to be considered in cases of post-ICI myelitis associated with paraneoplastic autoantibodies (such as CRMP-5 IgG). Patients treated with ICI have an underlying malignancy, and ICI can trigger immune responses against shared antigens between the tumor cells and the nervous system. Drug-induced myelopathies are therefore a heterogenous group of disorders, and the different pathophysiological processes that underlie these diseases should be considered when devising therapeutic strategies.

To summarize, all patients with suspected drug-induced myelitis should have comprehensive workup to rule out other causes including metabolic, neoplastic and infectious etiologies. Testing for AQP4, MOG and other neural antibodies, as well as CSF analysis including OCBs should be considered. Treatment of drug-induced myelitis should include discontinuation of the offending agent. Although this could be sufficient in mild cases of TNFI associated myelitis, most patients with drug-induced myelitis require additional treatment with intravenous corticosteroids. IVIG and plasmapheresis can be added in severe cases. For refractory cases, additional aggressive immunosuppressive agents could be considered. Long-term treatment is usually not required in drug-induced myelitis given monophasic disease course in most cases, although cases with a progressive or relapsing disease course may require prolonged immunosuppression. The choice of immunosuppressive agent should take into consideration the presence of specific pathogenic autoantibodies and/or the diagnosis of a specific underlying neuro-inflammatory condition such as MS, NMOSD or MOGAD. In addition, the choice of long-term immunosuppressive therapy should also consider the underlying disease process, i.e., the systemic inflammatory disorder or malignancy that the initial treatment was prescribed for. Generally, the offending drug should not be reinitiated after severe neurological complications have occurred. Given the association with worsening of demyelinating disease, TNFIs should be avoided in patients with a pre-existent diagnosis of a demyelinating disorder (40). While a pre-existent demyelinating disorder is not a formal contraindication for the use of ICIs, treatment decisions should be based on a thorough risk-benefit evaluation in the individual case (97, 99).

The increasing use of novel biological agents in the treatment of human diseases has led to a rise in iatrogenic CNS inflammation. While rare, drug-induced inflammatory myelopathies may be severe and a significant cause of disability for the patient. Early recognition and treatment is crucial to limit nervous system damage and the degree of residual symptoms. Neuroimmunologists, general neurologists and the providers prescribing these drugs should therefore be aware of the clinical presentation, diagnostic findings and management of these conditions.

## Author contributions

DG: acquisition and analysis of data and drafting the manuscript. CV-S: analysis of data, critical review, and editing the manuscript. All authors contributed to the article and approved the submitted version.

## Conflict of interest

The authors declare that the research was conducted in the absence of any commercial or financial relationships that could be construed as a potential conflict of interest.

## Publisher's note

All claims expressed in this article are solely those of the authors and do not necessarily represent those of their affiliated organizations, or those of the publisher, the editors and the reviewers. Any product that may be evaluated in this article, or claim that may be made by its manufacturer, is not guaranteed or endorsed by the publisher.
