# Supplementary material for: Drug-related immune-mediated myelopathies
Source: Front Neurol. 2022 Sep 29;13:1003270. doi: 10.3389/fneur.2022.1003270 (PMC9557103; doi:10.3389/fneur.2022.1003270)
Supplement: Supplementary file 1 [file Data_Sheet_1.docx]

**Supplementary Table 1: Reported cases of myelitis after tumor necrosis factor alpha inhibitors (TNFI)**

| **Author** | **Age**  **Sex** | **TNFI** | **Disease** | **Exposure time** | **Symptoms** | **MRI spine T2** | **MRI spine Gad** | **Brain MRI** | **CSF pleo-cytosis** | **CSF OCBs** | **Neural Ab** | **Treatment** | **Recovery** | **Rheum outcome & treatment** | **Other** |
| --- | --- | --- | --- | --- | --- | --- | --- | --- | --- | --- | --- | --- | --- | --- | --- |
| Lommers | 40  M | ADM | PS | 18 months | sensory | LETM, bulbo-medullary | + | normal | + | - | MOG-IgG | IVMP | complete | PS flare -photodynamic therapy | ON 17 mo later (MOGAD) - MMF |
| Yokoyama | 64  F | ETC | RA | 47 months | S/M | T8–9 | NR | normal | - | - | NR | IVMP | complete | RA flare - TOC |  |
| Defty | 48  F | ETC | RA | 4 years | sensory, bowel | C3-T1 | NR | normal | - | - | NR | MP | none | NR |  |
| Al Saieg | 58  F | ETC | RA | 1 year | S/M | C3-4 | NR | Demyelinating lesions | + | + | NR | IVMP | complete | NR | New MS diagnosis |
| Escalas | 40  F | ETC | PS | 21 months | S/M | C3 | + | normal | - | NR | NR | IVMP | complete | PS flare - ustekinumab |  |
| Finke | 55  F | ETC | PSA | 6 months | sensory, bladder | T | + | normal | - | NR | NR | IVMP | complete | No treatment (remission) |  |
| Sarathchandran | 59  F | ETC | RA | 8 years | S/M, bladder | LETM, C4-7 | + | normal | NR | NR | - | IVMP | partial | TOC for RA |  |
| Vadikolias | 40  M | INX | CD | 4 months | S/M | LETM, T9-12 | + | normal | + | - | NR | IVMP | partial | Ciprofloxacin & oral prednisone for CD |  |
| Barreras | 52  F | ADM | RA | 3 years | S/M | Multifocal, C2-T1 | - | normal | - | - | NR | IVMP | partial | Analgesics for RA |  |
|  | 52  F | ADM | CD | 1 year | S/M | LETM, C7-conus | + | normal | + | + | - | IVMP | partial | Oral prednisone for CD |  |
|  | 42  M | ADM | RA | 3 years | sensory | C5 | + | normal | + | + | NR | IVMP | none | MTX for RA, continued pain |  |
|  | 54  F | CTZ | RA+SLE | 2 years | sensory | T7 | - | normal | - | + | - | IVMP | partial | No treatment |  |
| Solomon | 51  F | ADM | CD | 2 years | S/M, bladder | normal | NR | nonspecific WM lesions | NR | NR | NR | None | none | NR |  |
|  | 61  F | INX | necrobiosis lipoidica | 9 months | sensory, bladder | T8-9 | NR | nonspecific WM lesions | + | - | NR | IVMP | partial | NR |  |
|  | 61  F | ETC | PS | 6 years | sensory, bladder | C-T | - | Demyelinating lesions | + | NR | NR | None | partial | PS worsened, no treatment | New MS diagnosis (relapse-IFN-SPMS) |
|  | 44  F | INX | AS | 18 months | motor | LETM, T9-12 | - | normal | - | NR | - | None | partial | No treatment |  |
| Theibich | 48  M | INX | AS | 17 months | sensory | multifocal, T2+C5 | NR | Demyelinating lesions | + | - | NR | RTX | partial | NR | New MS diagnosis |
| Fromont | 49  F | ETC | RA | 11 months | sensory | T11 | - | Demyelinating lesions | - | - | NR | None | none | RA flare – MTX & HCQ | New MS diagnosis (relapse-IFN) |
| Davis | 53  M | ETC | PSA | 6 months | NR | C-T | NR | NR | - | - | NR | None | none | NR | New MS diagnosis (relapse-IFN) |
|  | 51  F | ETC | AS | 18 months | NR | LETM | NR | Demyelinating lesions | - | NR | NR | None | partial | NR | New MS diagnosis |
| Kameda | 66  F | ETC | RA | 2 years | motor | LETM, C3-7 | NR | WM lesions | - | NR | NR | IVMP | partial | NR |  |

Abbreviations: Ab: antibodies, ADM: adalimumab, AS: ankylosing spondylitis, CD: Crohn’s disease, CSF: cerebrospinal fluid, C-T: cervico-thoracic, CTZ: certolizumab, ETC: etanercept, F: female, HCQ: hydroxychloroquine, IFN: interferon beta, INX: infliximab, IVMP: intravenous methylprednisolone, LETM: longitudinally extensive transverse myelitis, M: male, MMF: mycophenolate mofetil, mo: months, MRI; magnetic resonance imaging, MS: multiple sclerosis, NR: not reported, OCB: oligoclonal bands, ON: optic neuritis, PS: psoriasis, PSA: psoriatic arthritis, RA: rheumatoid arthritis, RTX: rituximab, SLE: systemic lupus erythematosus, S/M: sensory/motor, SPMS: secondary progressive MS, TOC: tocilizumab, WM: white matter

**Supplementary Table 2: Reported cases of myelitis after immune checkpoint inhibitors (ICI)**

| **Author** | **Age/**  **Sex** | **ICI** | **Cancer** | **Time to onset** | **Symptoms** | **MRI spine T2** | **MRI spine Gad** | **Brain MRI** | **CSF pleo-cytosis** | **CSF OCBs** | **Neural Ab** | **Treat-ment** | **Recovery** | **Recurrence myelitis & other irAE** |
| --- | --- | --- | --- | --- | --- | --- | --- | --- | --- | --- | --- | --- | --- | --- |
| Kunchok | 63  F | Atezo | SCLC | NR | S/M, bladder | LETM, C5-midT | + | NR | + | NR | CRMP5 | IVMP+ CTX | partial |  |
| Kubo | 69  M | Durva | NSCLC | 4 wk | S/M, bladder | LETM, T5-8 | NR | NR | - | NR | NR | IVMP | partial |  |
| O'Kane | 58  M | Ipi | Melanoma | 26 wk | motor | LETM, T7-L1 | NR | NR | + | NR | NR | IVMP | none |  |
| Liao | 62  M | Ipi | Melanoma | 7 wk | S/M, bladder/ bowel | T9-10 | NR | NR | + | NR | - | IVMP | partial | Uveitis, dermatitis, colitis, renal failure |
| Chang | 68  M | Ipi + Nivo  Pembro | Melanoma | 2 wk | sensory, bladder/ bowel | LETM, T5-10 | + | New mets | - | - | - | IVMP +PLEX, CTX, INX | none | Progressive myelitis |
| Carausu | 68  M | Pembro | NSCLC | 24 wk | S/M, bladder/ bowel | T12-L1 | + | NR | - | NR | - | PO steroids | complete |  |
| Garcia | 39  F | Ipi | Melanoma | 7 wk | motor, bladder/ bowel | LETM, C-T | + | LM Gad+ ,  T2-hyper right brachium pontis | + | NR | NR | IVMP, IVIG, INX | partial | Recurrence  Hypophysitis |
| Abdallah | 45  F | Ipi | Melanoma | NR | NR | Multifocal | Nerve  root | NR | + | NR | NR | PO steroids, INF | partial |  |
| Mancone | 64  NR | Ipi + Nivo | Melanoma | 40 wk | NR | NR | NR | NR | + | NR | NR | IVMP | partial |  |
| Narumi | 75  M | Nivo | NSCLC | 8 wk | motor, bladder/ bowel | LETM, C5-L1 | + | NR | + | - | AQP4-IgG | IVMP, PLEX | none |  |
| Wilson | 35  M | Pembro | HL | 4 wk | S/M, bladder/ bowel, hiccups/ vomiting | LETM, pons-T | NR | T2-hyper pons | + | NR | - | IVMP, IVIG, PLEX | partial |  |
| Shimada | 63  F | Pembro | NSCLC | 2 wk | NMOSD | LETM, C4-T1 | + | NR | + | NR | AQP4-IgG | IVMP, PLEX | partial |  |
| Esechie | NR | Atezo | SCLC | NR | S/M, bladder | LETM, C7-T7 | + | NR | NR | NR | NR | IVMP, PLEX | minimal |  |
| Moodie | 68  M | Durva | NSCLC | 12 mo | S/M, bladder | LETM C4-T11 | + | Non-specific | + | - | - | IVMP, PLEX, CTX, MTX | minimal | Recurrence |
| Wang | 70  F | Durva | SCLC | 4 mo | S/M, bladder | LETM, C47-T3 | - | stable mets | - | NR | MOG (1:10), CV2, SOX1, ZIC4 | IVMP, IVIG, PLEX | partial |  |
| Picca | 58  M | Ipi + Nivo | Melanoma | 4 mo | S/M, bladder | LETM, C-T | + | NR | + | - | unclassified | IVMP, PLEX, TOC, RUX | partial |  |
|  | 57  M | Nivo | NSCLC | 12 cyc | S/M, bowel | Multifocal | + | - | + | + | unclassified | PO steroids | minimal | Recurrence |
|  | 62  F | Nivo | NSCLC | 7 cyc | S/M, bladder/ bowel | LETM | + | stable mets | NR | NR | NR | IVMP | NR |  |
|  | 16  F | Pembro | Mesenteric IMT | 19 cyc | S/M, bladder | Multifocal | + | - | - | NR | NR | IVMP | complete |  |
|  | 59  M | Pembro | NSCLC | 5 cyc | S/M, bladder, dysphagia, altered consciousness | LETM, C1-T10 | LM | Peri-ventricular Gad+ | + | NR | GFAP | IVMP, PLEX | partial |  |
|  | 61  F | Pembro | NSCLC | 5 cyc | S/M | C3-C4 | - | stable mets | + | + | NR | IVMP | partial |  |
|  | 57  M | Nivo | NSCLC | 51 cyc | S/M, bladder | Multifocal | Caudal roots | Peri-ventricular lesions | normal | + | NR | IVMP | partial | Recurrence |
| Charabi | 63  M | Pembro | NSCLC | 3 wk | motor, bladder/ bowel | LETM, C-T | + | - | + | - | - | IVMP | partial | Recurrence  Enteritis |
| Bolz | 55  M | Ipi | Melanoma | 8 wk | S/M | C1, C3-5 | NR | negative | + | NR | - | IVMP | partial |  |
| Nowosielski | 47  M | Ipi | Melanoma | 9 wk | S/M, bladder/ bowel | LETM | + | Peri-ventricular lesions Gad+ | + | negative | - | IVMP  RTX | complete | Recurrence  Arthritis |
| Poretto | 73  M | Nivo | RCC |  | sensory, bladder | LETM, T3-conus | + | new mets | - | NR | - | IVMP | minimal |  |
| Vickers | 73  M | Pembro | Bladder | 9 wk | S/M | - | - | - | + | NR | NR | IVIG | partial |  |
| Brahmbhatt | 61  F | Ipi + Nivo | Melanoma | NR | S/M | LETM | + | NR | + |  | - | IVMP | complete | Recurrence |
| Makkawi | 53  F | Nivo | Melanoma | 10 mo | S/M, bladder | LETM, T1-12 | + | NR | + | + | - | IVMP | partial | Recurrence |
|  | 54  F | Ipi + Nivo | Melanoma | 14 wk | S/M, bladder | LETM, C2-conus | + | NR | + | + | - | IVMP, PLEX | complete | Recurrence |

Abbreviations: Ab: antibodies, Atezo: atezolizumab, CTX: cyclophosphamide, C-T: cervico-thoracic, CSF: cerebrospinal fluid, cyc: cycles, Durva: durvalumab, F: female, Gad+: contrast enhancing, HL: Hodgkin lymphoma, IMT: inflammatory myofibroblastic tumor, INX: infliximab, Ipi: ipilimumab, IVIG: intravenous immunoglobulin, IVMP: intravenous methylprednisolone, irAE: immune-related adverse events, LETM: longitudinally extensive transverse myelitis, LM:leptomeningeal, M: male, mets: metastases, mo: months, MRI; magnetic resonance imaging, MS: multiple sclerosis, MTX: methotrexate, Nivo: Nivolumab, NMOSD: neuromyelitis optic spectrum disorder, NR: not reported, NSCLC: non-small cell lung cancer, OCB: oligoclonal bands, Pembro: pembrolizumab, PLEX: plasmapheresis, RCC: renal cell carcinoma, RTX: rituximab, RUX: ruxolitinib, SCLC: small cell lung cancer, S/M: sensory/motor, TOC: tocilizumab, T2-hyper: T2-hyperintensity, wk: weeks, WM: white matter
